# Cross-species identification of in *silico* microsatellite biomarkers for genetic disease

**DOI:** 10.7603/s40681-014-0014-1

**Published:** 2014-08-02

**Authors:** Hao-Teng Chang, Yu-Yang Lo, Jhen-Li Huang, Wei-Yong Lin, Tun-Wen Pai

**Affiliations:** 1 Graduate Institute of Basic Medical Science, China Medical University, Taichung, Taiwan; 2 Department of Computer Science and Information Engineering, Asia University, Taichung, Taiwan; 3 Department of Computer Science and Engineering, National Taiwan Ocean University, Keelung, Taiwan; 4 Graduate Institute of Integrated Medicine, China Medical University, Taichung, Taiwan; 5 Department of Computer Science and Engineering & Center of Excellence for the Oceans, National Taiwan Ocean University, No. 2, Peining Road, Keelung, 20224 Taichung, Taiwan

**Keywords:** Microsatellite, Simple sequence repeat, Genetic disease

## Abstract

Microsatellites appear widely in genomes of diverse species. Variants of repeat number of microsatellites often correlate with risks of genetic disorder or severity of diseases. Using cross-species comparison, the proposed system comprehensively verifies microsatellites of specific genes related to 16 genetic disorders. Genomic information retrieved from 14 frequently used model organisms in biomedical study was thoroughly analyzed, emphasizing conserved and diverse traits. Features of microsatellite sequences among different organisms, including appearing frequency, position, pattern and distribution, could be determined automatically for stating genetically functional conservation and evolutionary correlation. This research found that among mammals and fishes, the microsatellite sequences are conserved in the genes of epidermal growth factor receptor, ataxia telangiectasia mutated and androgen receptor corresponding to cancers, ataxia telangiectasia and hepatocellular carcinoma, respectively. Still, except fruit fly conserved CAG repeats in Huntington and Spinocerebellar ataxia type 2 genes, no microsatellites were conserved in those genes linked to neurological/neurodegenerative disorders among mammal and fish species. In comparison of mammalian species, microsatellite biomarkers identified from 17 genetic disorder-related genes revealed high repeat conservation, especially in human, gorilla and macaque. Obviously, this comparative analysis illustrates microsatellite repeats affecting genetic disorders, highly correlated to evolutionary distance of species. Chief contribution of this *in silico* research lies in assisting biologists to identify disease-related microsatellite biomarkers and employ appropriate model organisms for further biomedical studies relying on microsatellite conservation information. Database http://ssrtc.cs.ntou.edu.tw is for academic use.

Microsatellites, also known as simple sequence repeats (SSRs), are patterns existing in a broad gamut of species, accounting for 2% of total human genome [1]. In general, basic repeat length consists of from one to six nucleotides [2]. Different from other kinds of biomarkers, microsatellites evolve rapidly, distribute widely and escape natural selection, making them ideal molecular biomarkers for identification testing, paternity testing, and criminal investigation, based upon their advantages of various polymorphisms and simple detection using polymerase chain reaction [3, 4]. Recent reports mentioned that in certain genes, length or position of microsatellites would alter regulation of biological functions directly or indirectly [5]. Copy number or sequence variation of microsatellites may cause genetic disorders like Huntington’s chorea, a severe neurodegenerative genetic disorder arising from abnormal increase of “CAG” triplet repeats in Huntington (HTT) gene coding region in chromosome 4 [6]. Multiple “CAG” repeats in HTT gene encodes poly-glutamine (poly-Q) peptides trigger cytotoxic protein misfolding and aggregation in neurons, which impair muscle coordination, lead to cognitive decline and psychiatric problems, finally causing death from respiratory failure.

Microsatellites also play a crucial role in species evolution. Repeat numbers of microsatellites usually presented resources of variations in morphological evolution [7]. Yet it is difficult to select useful, functional microsatellites for biological studies or clinical investigation owing to myriad patterns embedded in large amount of repeats. Also, conservation of microsatellites derived from various species was still unclear. It is thus important to develop an effective and efficient tool for identifying functional microsatellites and analyzing conserved and exclusive features of cross-species microsatellites. We designed an algorithm to ferret out microsatellite candidates with highly conserved traits via cross-species comparison or unique motif patterns apparent in certain group of genes or organisms. Selected species genomes contain two major categories: mammals (*Mus musculus, Canis familiaris, Bos Taurus, Macaca mulatta, Gorilla gorilla, Homo sapiens*) and fish (*Danio rerio, Gasterosteus aculeatus, Oryzias latipes, Gadus morhua, Tetraodon nigroviridis, Takifugu rubripes*), plus two common model organisms of fruit fly (*Drosophila melanogaster*) and roundworm (*Caenorhabditis elegans*). Genomic sequences and gene annotation of these selected model organisms were retrieved from Ensembl database version 65, which contain information on sequence IDs and positions of coding, exon, intron and 5’-/3’-untranslated region (UTR) of each gene. Microsatellites were highly variable; repeat variations likely caused severe disorders. To ascertain whether mining of microsatellite biomarkers could be achieved by cross-species comparison, 16 genetic inheritance diseases originating from microsatellite repeat variations were incorporated into this study (Table 1) [8].

To expedite identification of orthologous microsatellites from various species, all microsatellite candidates were pre-identified by auto-correlation search algorithms [9]. Length of microsatellite in this study was defined as equal to or greater than twenty nucleotides, basis repeat pattern of each microsatellite comprised one to six nucleotides. Still, during DNA synthesis and replication, genetic variation (insertion, deletion, substitution) can yield imperfect microsatellite repeat patterns. We defined the variant types as noise in imperfect microsatellites. Except for identification of perfect microsatellite patterns, our algorithms allowed comparison on microsatellite repeats containing multi-scale noise by setting tolerance parameters.

Position and length of microsatellites within each gene were identified, analyzed, noted, recorded and stored in the designed microsatellite database. According to the gene annotation and coordination defined by Ensembl, positions of microsatellites were also stored. Users define a set of genes, groups of interesting species genomes, and specific patterns of microsatellite sequence as per their requests. The proposed system can automatically compare and analyze occurrence frequency and differences of microsatellites. Output information comprises repeat pattern, length, genetic loci within genomes, and conserved and exclusive levels of microsatellites among species, whose information can serve as an indicator for selecting model organisms in studies of microsatellite-related diseases.

Microsatellites inter-dispersively distribute in 7 areas of a gene including coding region, exon, intron, 5’-untranslated region (UTR), 3’-UTR, upstream and downstream. To limit extent of the query gene, the regions of upstream and downstream were extended to 2,000 nucleotides at upstream of 5’-UTR and downstream of 3’-UTR, respectively. Based on shifting mechanisms, different patterns of microsatellites might be recognized as the same ones. For example, within a piece of DNA sequence “ACTACTACTACT”, repeat patterns of “ACT”, “CTA” or “TAC” could be defined as the same repeat unit. Also, with DNA sequence complementary, those complementary patterns also would be recognized as the same. This definition recognizes 501 possible patterns as distinct microsatellite repeats [10].

As mentioned, coding regions are relatively important for determining alteration of protein functions due to poly-residue insertion caused by multiple microsatellite repeats. The proposed system translates coding sequences into amino acid residues so that biologists can readily observe mutations within genes. To describe position of microsatellites, the system designed novel presentation method composed of repeat pattern and its corresponding position: e.g., “ACC@Coding” means a microsatellite composed of multiple ACC repeats, motif located

at coding region of query gene with length greater than 20 nucleotides. Since microsatellites distribute widely in species genomes, biologists meet obstacles in searching and identifying bio-functional microsatellites. This proposed system allows users to set microsatellite conservation ratio that provides information of conservation degrees of microsatellites within a specified species group. Users can define species clusters in advance according to research purpose; cross-species comparison identifies ortholog microsatellites.

Analysis of cross-species microsatellites identified from 16 genetic disorders plotted correlation between genetic disorder and phylogenic relationship. Among the microsatellites of genetic disorders, mammals conserved most. Disease-related types include epidermal growth factor receptor (EGFR) for cancer, Ataxia telangiectasia mutated (ATM) for Ataxia telangiectasia and Androgen receptor (AR) for liver cancer conserved in both mammals and fish (Table 1). By comparing microsatellites of 16 human genetic disorders, we observed higher microsatellite conservation degrees in closer species. This gave implications and practical applications: with conservation degrees of microsatellites among species, biologists choose appropriate and rational research model organisms for probing microsatellite-related genetic disorders.

Since mice are the most common model organisms in biomedical research, we compared identified microsatellites within genetic disease genes for both human and mouse genomes. We spotted microsatellite repeats in FMR1, EGFR, and X25 (Table 1). FMR1 gene contains GCG repeats at 5’-UTR whose site served as a CpG island for regulating transcription activities [11, 12]. What with similar regulation of FMR1 expression between mice and humans, identities of DNA and amino acid sequences of FMR1 are 95% and 97%, respectively. Regulatory as well as coding regions of FMR1 are well conserved in humans and mice during evolution. Likewise, CA microsatellite repeats in intron region of EGFR are also conserved well among species of mammals and fishes. It might indicate zebrafish, et al. as ideal model organisms for studying EGFR-related diseases.

While mice are well-established as the most popular animals for biomedical or disease research, time and money expended pose disadvantages to any such model. According to our genomic analysis, fruit flies reserves CAG repeats at coding regions in HTT and SCA2 genes, corresponding to Huntington disease and Spinocerebellar ataxia, respectively. Fruit flies may prove genetically adequate for studying these two diseases. CAG repeats engender poly-glutamine sequences in certain proteins that usually affect neurodegenerative disorder: e.g., Huntington’s chorea, Spinocerebellar ataxia, Parkinsonism. Because fruit flies possess complete brains, eyesight and ability for learning and memory, yet exhibit a life span relatively short while experimental budget is relatively low, it could be suggested as a powerful tool for investigating the microsatellite-related neurodegenerative disorders [13].

We herein identify microsatellites from clustered multiple species to establish a microsatellite database that can present repeat position, pattern and conservation degree within a gene. Furthermore, we analyze disease-related microsatellites to find these well conserved among several species, either mammal or fish species clusters. This study proposed that microsatellites might relate to the evolutionary event; their conservation might yield another rationale for lower organisms’ use in disease study.

**Table 1:** Cross-species comparison between mammals and fishes.

Gene^a^	GID	Disease	Repeat pattern@region	Cross-species compa	
				Mammals	Fishes
DMPK	ENSG	Myotonic dystrophy type 1	CTG@3UTR [11]	Human	Non
	00000104936			Gorilla	
ATN1	ENSG	Dentatorubral-pallidoluysian atrophy (DRPLA)	CAG@Coding [12]	Huamn	Non
	00000111676			Macaca	
				Dog	
EGFR	ENSG	Cancers	CA@Intron [13]	Cow	Zebrafish
	00000146648			Dog	Stickleback
				Macaca	Fugu
				Mouse	Tetraodon
				Huamn	Cod
				Gorilla	
ATM	ENSG	Ataxia-telangiectasia	T@Intron [14]	Dog	Zebrafish
	00000149311			Macaca	
				Huamn	
				Gorilla	
AR	ENSG	Cancers	CAG@Coding [15]	Dog	Stickleback
	00000169083			Macaca	
				Huamn	
				Gorilla	
HTT	ENSG	Huntington’s Disease	CAG@Coding [16]	Cow	Non
	00000197386			Human	
FMR1	ENSG	Fragile X syndrome	GCG@5UTR [17]	Mouse	Non
	00000102081			Human	
FMR2	ENSG	Fragile XE syndrome	GCC@5UTR [18]	Human	Non
	00000155966				
C9orf72	ENSG	Amyotrophic lateral sclerosis ALS)/ Frontotemporal dementia (FTD)	GGGGCC@Upstream [19]	Human	Non
	00000147894				
X25^b^	ENSG	Friedreich ataxia	GAA@Intron 1 [20]	Macaca	Non
	00000165060			Mouse	
				Human	
SCA1	ENSG	Spinocerebellar ataxia type 1	CAG@Coding [21]	Dog	Non
	00000124788			Macaca	
				Huamn	
				Gorilla	
SCA2	ENSG	Spinocerebellar ataxia type 2	CAG@Coding [21]	Cow	Non
	00000204842			Human	
SCA3	ENSG	Spinocerebellar ataxia type 3 (Machado-Joseph disease)	CAG@Coding [21]	Macaca	Non
	00000066427			Human	
SCA6	ENSG	Spinocerebellar ataxia type 6	CAG@Coding[21]	Human	Non
	00000141837				
SCA7	ENSG	Spinocerebellar ataxia type 7	CAG@Coding [21]	Dog	Non
	00000163635			Human	
SCA12	ENSG	Spinocerebellar ataxia type 12	CAG@5UTR [22]	Macaca	Non
	00000156475			Human	

^a^ Setting of parameters for all genes excluding X25: Conserved ratio: 80%. Tolerance: 0%.

^b^ Setting of X25 gene: Conserved ratio: 80%. Tolerance: 20%.

## Acknowledgments

This work is funded by the Center of Excellence for the Oceans of National Taiwan Ocean University and National Science Council, Taiwan, R. O. C. (NSC102-2321-B-019-001 to T.-W. Pai; NSC102-2622-B-039-CC3 and NSC102-2628- B-039-008- MY3 to H.-T. Chang), and in part the Department of Health in Taiwan (DOH102-TD-B-111-004).
